# Glycopatterns of Urinary Protein as New Potential Diagnosis Indicators for Diabetic Nephropathy

**DOI:** 10.1155/2017/5728087

**Published:** 2017-03-19

**Authors:** Hanyu Zhu, Moyan Liu, Hanjie Yu, Xiawei Liu, Yaogang Zhong, Jian Shu, Xinle Fu, Guangyan Cai, Xiangmei Chen, Wenjia Geng, Xiaoli Yang, Minghui Wu, Zheng Li, Dong Zhang

**Affiliations:** ^1^Department of Nephrology, Chinese PLA General Hospital, Chinese PLA Institute of Nephrology, State Key Laboratory of Kidney Diseases, National Clinical Research Center of Kidney Diseases, Beijing Key Laboratory of Kidney Disease, Beijing, China; ^2^Department of Nephrology, General Hospital of Jinan Military Command, Jinan, China; ^3^Laboratory for Functional Glycomics, College of Life Sciences, Northwest University, Xi'an, China

## Abstract

Diabetic nephropathy is a major cause of chronic kidney disease and end-stage kidney disease. However, so little is known about alterations of the glycopatterns in urine with the development of diabetic nephropathy. Presently, we interrogated glycopatterns in urine specimens using a lectin microarray. The results showed that expression levels of Sia*α*2-6Gal/GalNAc recognized by SNA exhibited significantly increased tendency with the development of diabetic nephropathy; moreover, SNA blotting indicated glycoproteins (90 kDa, 70 kDa, and 40 kDa) in urine may contribute to this alteration. Furthermore, the glycopatterns of (GlcNAc)_2–4_ recognized by STL exhibited difference between diabetic and nondiabetic nephropathy. The results of urinary protein microarray fabricated by another 48 urine specimens also indicated (GlcNAc)_2–4_ is a potential indictor to differentiate the patients with diabetic nephropathy from nondiabetic nephropathy. Furtherly, STL blotting showed that the 50 kDa glycoproteins were correlated with this alteration. In conclusion, our data provide pivotal information to monitor the development of diabetic nephropathy and distinguish between diabetic nephropathy and nondiabetic renal disease based on precise alterations of glycopatterns in urinary proteins, but further studies are needed in this regard.

## 1. Introduction

Diabetic nephropathy (DN) is a serious complication of diabetes mellitus and now the major cause of chronic kidney disease (CKD) and end-stage kidney disease (ESRD) throughout the world. However, renal damage associated with diabetes can be classified as either DN or nondiabetic renal disease (NDRD). There are significant differences in the treatment and prognosis of these two types of renal disease. Renal pathological diagnosis is currently the gold standard for distinguishing DN from NDRD, although renal biopsy is invasive.

Urine has some unique advantages (collected noninvasively, a large number of proteins in urinary proteome also belong to plasma proteome); certain proteins in urine not only reflect the status of the entire body, but also correlated with diseases, which is good resource for the analysis of disease processes [1–4]. Valuated urinary proteome analysis is a tool for prediction and monitoring of DN. Transferrin is a glycoprotein with a molecular weight of 76.5 kDa, which was demonstrated that the urinary transferrin could reflect the degree of interstitial fibrosis and tubular atrophy in patients with DN [5]. The low-molecular weight proteome in urine was profiled by capillary electrophoresis-coupled mass spectrometry (CE-MS), and results indicated that the specific collagen fragments in urine could effectively assess DN risk at an early stage [6]. For type 2 diabetic nephropathy, it is reported that urinary cystatin C and NAP (nonalbumin protein) could reflect the progression of type 2 diabetic nephropathy [7]. Liver-type fatty acid-binding protein (L-FABP), an intracellular carrier protein of free fatty acids, is expressed in the liver and kidney, which could serve as a predictive marker for renal and cardiovascular prognosis in type 2 diabetic patients without advanced nephropathy [8]. It is interesting that L-FABP also could be an independent predictor of progression at all stages of type 1 diabetic nephropathy irrespective of stage [9].

Glycosylation is an important posttranscription modification; more than half of known proteins are glycoprotein. It is well known that the glycan attached on glycoproteins has many biological functions, such as maintaining conformation and flexibility of protein, oligosaccharide modification involved in biological processes including cell adhesion, signal transduction, and endocytosis. Abnormal glycosylation had demonstrated correlation with occurrence and development of diseases [10, 11]. Lectin microarray is a powerful technology that uses a panel of lectins immobilized on solid phase substrate for high-throughput analysis of glycans and glycoproteins related to diseases such as cancer and inflammatory diseases [12–14]. Alterations of sialylated MUC1 glycosylation detected by lectin microarray could be indispensable for the development of cholangiocarcinoma [15]. Glycosylated patterns of A1AT (alpha-1-antitrypsin) may serve as potential biomarkers for early detection [16]. For DN, nonenzymatic glycosylation product such as glycated albumin was strongly associated with incident diabetes and its microvascular complications. In addition, the glycated albumin: HbA1c ratio and glycated albumin may be superior to HbA1c associated with the presence of DN [17, 18]. However, there were few reports about enzymatic glycosylation on glycoproteins that altered in development of DN.

In this study, we preformed lectin microarray to investigate altered glycopatterns during the development of DN and evaluate whether glycopattern in urine was a potential biomarker to differentiate DN from NDRD.

## 2. Materials and Methods

### 2.1. Study Population

Our study subjects were patients who underwent renal biopsy at the Chinese People's Liberation Army (PLA) General Hospital. The inclusion criteria were as follows: patients aged between 20 and 70 years; patients diagnosed with type 2 diabetes mellitus (type 2) and with persistent overt proteinuria (defined as urinary albumin excretion ≥ 300 mg/24 h or urinary protein excretion ≥ 500 mg/24 h by at least two tests without evidence of urinary tract infection); patients with serum creatinine less than 442 *μ*mol/L; and patients who were voluntarily admitted to the hospital to undergo renal biopsy. The exclusion criteria were the following: patients who had a confirmed diagnosis of renal diseases before they were diagnosed with type 2 diabetes; with a clinically confirmed diagnosis of NDRD, including lupus nephritis and Henoch-Schönlein purpura nephritis; with familial hereditary nephropathies, such as autosomal dominant polycystic kidney disease; and with an uncertain pathological diagnosis.

The tissue was examined by at least two pathology experts together with another two nephrologists; then the pathological diagnosis was determined. Renal biopsy indications: in clinical practice, the possible diagnosis of NDRD was confirmed by renal biopsy when the following situations occurred: gross/microscopic hematuria; elevated serum creatinine without obvious proteinuria; persistent massive proteinuria with normal renal function and no diabetic retinopathy. The renal biopsy indications for the suspected diagnosis of NDRD at our centre were in accordance with those listed for NDRD in the 2007 Kidney Disease Outcomes Quality Initiative (KDOQI) guidelines. Thus, 19 cases were diagnosed as DN and 18 cases were diagnosed as NDRD which included 10 (membranous nephropathy) MN and 8 IgAN. To evaluate whether glycopattern in urine was a potential biomarker to differentiate DN from NDRD, the presence of other kidney diseases accompanied with DN or NDRD and the patients who had pathological diagnosis of DN combined with NDRD were excluded in this study.

In order to evaluate potential biomarkers for distinguishing DN from NDRD, all the patients were divided into DN and NDRD group. To assess the possibility of urinary glycopatterns as potential biomarkers for monitor the process of DN, we separate the DN group into two subgroups according to the eGFR level. The eGFR level was calculated using the Modification of Diet in Renal Disease (MDRD) Study formula. The DN group I defined as the eGFR > 60 mL/min/1.73 m^2^, along with low level 24 h urinary protein (1.41 ± 0.61 g/24 h), means the early stage of kidney disease. The DN group II defined as eGFR < 60 mL/min/1.73 m^2^, along with high level 24 h urinary protein (2.64 ± 1.41 g/24 h), means the patients have renal insufficiency, indicating a worse prognosis.

All healthy volunteers (HVs) had no recent illness or medication. All females had no menstrual cycle at the time of collection. The collection of urine samples was done prior to treatment and before the biopsy or at least 1 month after the biopsy. The clinical information was summarized in Table 1.

### 2.2. Study Approval

The collection and use of all human pathology specimens for research presented here were approved by the Ethical Committee of Northwest University (Xi'an, China), the Department of Nephrology, Chinese PLA General Hospital, Chinese PLA Institute of Nephrology, State Key Laboratory of Kidney Diseases, National Clinical Research Center of Kidney Diseases, Beijing Key Laboratory of Kidney Disease (Beijing, China). Written informed consent was received from participants for the collection of their urine. This study was conducted in accordance with the ethical guidelines of the Declaration of Helsinki.

### 2.3. Processing of Urine

15 mL of urine was collected and stored at 4°C immediately. Then, the samples were centrifuged at 3000 rpm for 20 minutes at 4°C to remove cells and debris. The supernatant was frozen at −80°C in 1 mL aliquots. The method of trichloroacetic acid (TCA)/acetone precipitation was utilized to prepare urinary protein; the method of urinary protein precipitation was described elsewhere with little modification [19–21]. Briefly, 3 mL mixed urine specimen was mixed with 30 mL TCA 10%-acetone solution 90% (v/v), followed by overnight incubation at −20°C. The mixture was centrifuged at 10,000*g* for 15 min at 4°C; after that, protein precipitate was resuspended by 2 mL ice-cold acetone; after centrifugation, the protein was left to dry on ice for 1 h. Pellet resuspension was performed with 500 *µ*L 10 mM PBS buffer (pH 7.4) with 1% Tween-20 at ambient temperature for 15 min, sonicated for another 15 min in ultrasound bath. The protein concentration was determined by Bradford assay (Sigma-Aldrich, St. Louis, MO).

### 2.4. Lectin Microarrays

**Figure 1:**
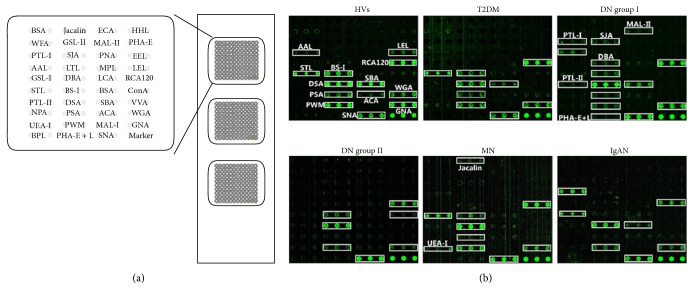
Glycopatterns of urine glycoproteins from HVs, patients with T2DM, DN group I, and DN group II by lectin microarrays. (a) The layout of the lectin microarray. Each lectin was spotted in triplicate on one slide. Cy3-labeled BSA was spotted as a location marker and BSA as a negative control. (b) The profile of Cy3-labeled urinary proteins from HVs; patients with T2DM, DN group I, DN group II, MN, and IgAN bound to the lectin microarrays, respectively. The lectins showing the effective data were marked with white frames.

To reduce the differences between subjects and to tolerate individual variation, urinary protein was pooled equivalently according to sample classification, and each class was divided into three biological replications. The urinary proteins (80 *µ*g) were mixed with equal volume of Na_2_CO_3_ solution (100 mM, pH 9.3) and then labeled with Cy3 fluorescent dye (GE Healthcare, Biosciences, Piscataway, NJ, USA); the mixture was incubated for 3 h at ambient temperature in darkness. After that, the labeled protein was purified with Sephadex G-25 columns (GE healthcare) according to the manufacturer's instructions to remove uncombined fluorescent dye. The lectin microarray was fabricated by using 37 exogenous plant lectins as purchased from Vector Laboratories (Burlingame, CA), from Sigma-Aldrich (St. Louis, MO), and from Calbiochem Merck (Darmstadt, Germany) with different binding preferences covering N- and O-linked glycans. The lectins were dissolved to a concentration of 1 mg/mL in the manufacturer's recommended buffer, which contained 1 mmol/L appropriate monosaccharide. Lectins were spotted on the homemade epoxysilane-coated slides with Stealth micro spotting pins (SMP-10B) (TeleChem, USA) by a Capital smart microarrayer (CapitalBio, Beijing). Please refer to Qin et al. [22] for the sugar-binding specificities of the lectins, and layout of the lectin microarray was showed in Figure 1(a). Each lectin was spotted in triplicate per slide. After that, the slides were incubated in a humidity-controlled incubator at 50% humidity overnight and then put in vacuum dryer for 2 h at 37°C to immobilize. Then, the slides were blocked with the blocking buffer containing 1% (w/v) BSA in 10 mM PBS (pH 7.4) for 1 h at 25°C and then rinsed twice with 1 × PBST (0.2% Tween-20 in 10 mM PBS, pH 7.4), followed by a final rinse in 10 mM PBS. Previously, we had determined that 4 *μ*g labeled protein was used to ensure that all binding signals were produced in the linear binding range of each lectin for the lectin microarrays [22]. Therefore, 4 *μ*g of Cy3-labeled protein mixed with 0.5 mL of incubation buffer containing 2% (w/v) BSA, 500 mM glycine, and 0.1% Tween-20 in 10 mM PBS was applied to incubate with the lectin microarrays, and an incubation was performed in the chamber at 25°C for 3 h in a rotisserie oven set at 4 rpm. After incubation, the slide was washed with 1 × PBST twice for each for 10 min and washed twice with 10 mM PBS for 5 min and then dried by centrifugation at 600 rpm for 5 min at room temperature. The microarrays were scanned with the 70% photomultiplier tube and 100% laser power settings using a GenePix 4000B Microarray Scanner (Axon Instruments, USA).

### 2.5. Data Acquisition and Analysis

The fluorescence signal intensity of each spot was extracted by GenePix 3.0 software (Axon Instruments, Inc., USA). To avoid the nonspecific adsorption, if the signal intensity of one lectin spot was less than average background + standard deviation (SD), it is regarded as invalid spot and removed. After data filtered, the median of the effective data points of each sample was counted. To eliminate fluorescence bias between different data sets, the fluorescence signals of each lectin microarray were treated by max-normalization [23–25]. Briefly, the signals of each lectin were normalized to the highest signal intensity among 37 lectins. After normalization, the processed data of the parallel data sets were compared with each other based upon fold changes according to the following criteria: fold changes ≥ 1.5 or ≤0.67 and *p* < 0.05 in the pairs indicated up- or downregulation of certain kind of glycopatterns, respectively. Differences between the arbitrary two data sets or multiple data sets were tested using Student's *t*-test or Kruskal-Wallis test; the histogram and ROC curve analysis were performed by GraphPad Prism 6. The data were further analyzed by Expander 6.4 (version 6.4; http://acgt.cs.tau.ac.il/expander/) in order to perform hierarchical clustering analysis; the unweighted pair group method was constructed with arithmetic-mean tree using Pearson's correlation as the metric of similarity. The Spearman rank-order coefficient (rs) was calculated by Spearman rank correlation analysis.

### 2.6. Urinary Protein Microarrays

In order to verify the results of the lectin microarrays, a urinary protein microarray was produced by using another 48 individuals urine samples; the detailed sample information was showed in Table S1 (see Supplementary Material available online at https://doi.org/10.1155/2017/5728087). The fabrication and incubation of urinary protein microarrays were similar with lectin microarrays. Briefly, urinary proteins were dissolved in spotting buffer containing 0.5 mg/mL BSA in 10 mM PBS (pH 7.4) to a concentration of 1 mg/mL and spotted on the homemade epoxysilane-coated slides with Stealth micro spotting pins (SMP-10B) by a Capital smart microarrayer. A urinary protein microarray was produced by spotting 48 individual urine samples (7 HVs, 9 T2DM patients, 15 patients with DN (7 DN group I, 8 DN group II), and 17 patients with NDRD (9 MN and 8 IgAN)) on the surface of the epoxy slide. Each urine specimen was spotted in triplicate per slide and incubated in a humidity-controlled incubator at 50% humidity overnight for immobilization. Then, the slides were blocked with the blocking buffer for 1 h; after washing, the blocked slide incubated with 4 *μ*g Cy3 labeled lectin diluted in 0.5 mL of incubation buffer for 3 h at room temperature in the dark. The slide was washed with 1 × PBST twice for each for 10 min and washed once with 10 mM PBS for 5 min and then dried by centrifugation at 600 rpm for 5 min. The slides were scanned with the 70% photomultiplier tube and 100% laser power settings using a GenePix 4000B Microarray Scanner and the signal intensity was extracted by GenePix 3.0 software.

### 2.7. SDS-PAGE and Lectin Blotting

Urinary glycoproteins were further analyzed using lectin blotting. Firstly, the pooled urinary proteins were analyzed by SDS-PAGE. Briefly, urinary proteins were mixed with 5 × loading buffer and boiled for 5 min at 100°C; after that, the samples were spun down and loaded on a 10% polyacrylamide resolving gel and a 3% stacking gel, and protein molecular weight marker (Thermo Scientific, Waltham, USA) was run in gel simultaneously. After being immobilized, the gel was stained by alkaline silver. For lectin blotting analysis, the proteins in gels were transferred to a PVDF membrane (Millipore, MA, USA) at 100 V for 90 min in wet transfer device (Beijing Liuyi Instrument Factory, China). Subsequently, the membranes were washed twice with TBST buffer (150 mM NaCl, 10 mM Tris-HCl, and 0.05% v/v Tween-20, pH 7.5) and blocked for 1 h with Carbo-Free Blocking Solution (Vector, Burlingame, CA) for 1 h at ambient temperature. The membranes were then incubated with 3 *µ*g/ml Cy5 (GE Healthcare, Biosciences, Piscataway, NJ, USA) labeled lectins in previous blocking solution with gentle shaking overnight at 4°C below protection from light. The membranes were washed twice each for 10 min with TBST and scanned by Storm 840 PhosphorImager (Molecular Dynamics, Sunnyvale, CA) in red fluorescence channel 635 nm excitation/650LP emission.

## 3. Result

### 3.1. Alterations of Urinary Glycopatterns with the Development of DN

About 30–40% of patients with type 1 or type 2 diabetes develop evidence of nephropathy, responsible for 40~50% of all ESRD. The glycopatterns in urine from HVs and patients were evaluated by the lectin microarrays. The glycopatterns of Cy3-labeled pooled urinary proteins from HVs and all patients bound to the lectin microarrays were shown in Figure 1(b). The normalized fluorescent intensities (NFIs) for each lectin were summarized as the mean values ± SD in Table S2.

**Figure 2:**
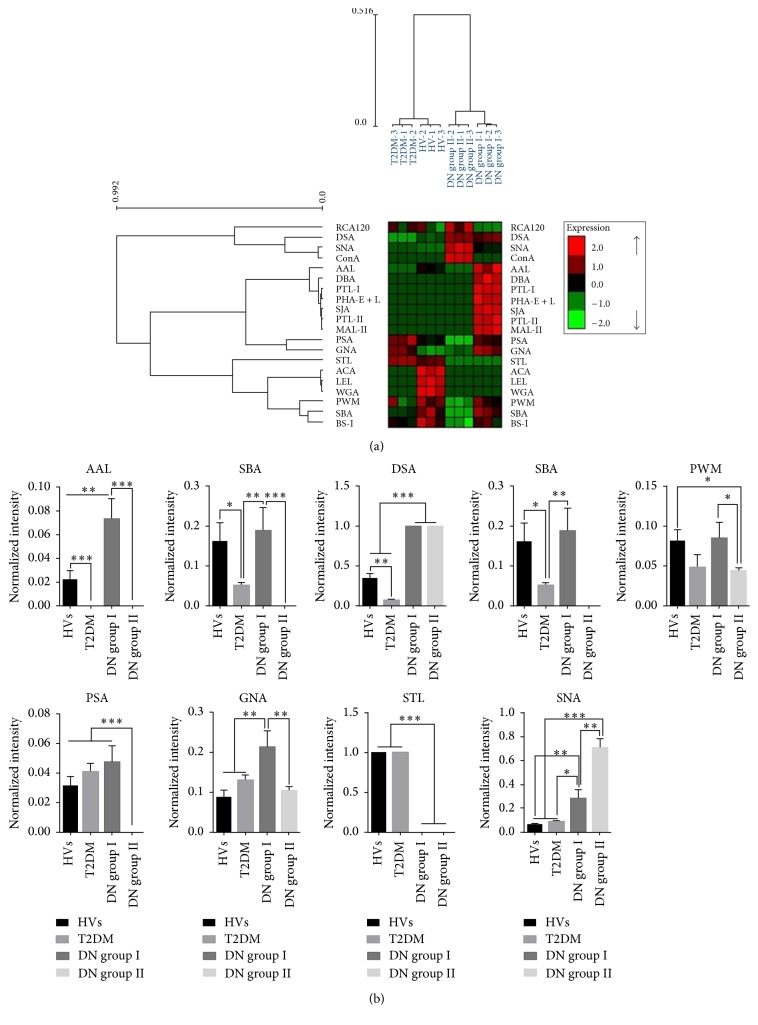
The alterations of urinary glycopatterns associated with development of DN. (a) Hierarchical clustering analysis of the 20 lectins with three biological replicates. Samples were listed in columns and the lectins were listed in rows. The color and intensity of each square indicated expression levels relative to other data in the row. Red: high, green: low, and black: medium. (b) Comparing the NFIs of the urinary glycopatterns based upon ratio of each lectin between HVs, T2DM, and DN groups. Significant differences between groups were analyzed according to Student's *t*-test, respectively (^*∗*^*p* < 0.05, ^*∗∗*^*p* < 0.01, and ^*∗∗∗*^*p* ≤ 0.001). The data were the averaged NFIs ± SD of three biological replicates.

Firstly, we investigated the glycopattern of urinary glycoproteins in HVs, patients with T2DM, and different degree of DN separately. The NFIs of three biological replicates were imported EXPANDER 6.4 for hierarchical clustering analysis (HCA). The NFIs values mapped to the range of 2 to −2 interval were color coded, with red and green indicating an increase and decrease in the abundance of glycopatterns, respectively. The HCA revealed that both DN groups were clustered into one class, whereas the HVs and T2DM clustered into another class, and lectins were categorized into three independent groups according to the NFIs of lectins showing the positive signals in HVs and patients (Figure 2(a)). The results indicated that the abundance of glycan patterns was different between HVs, T2DM, and DN groups. Further, the NFIs of each lectin from HVs and patients were compared based on fold change in pairs, with all* p* values below 0.05, to evaluate whether the glycopatterns of urinary glycoproteins were altered with the development of DN (Table 2). Compared the alterations of glycopatterns in urine between HVs and T2DM, the results showed that the *α*-Fuc binder AAL, LacNAc, poly-LacNAc and (GlcNAc)_n_ binder LEL, Gal*β*1-3GalNAc*α*-Ser/Thr (T-antigen) binder ACA, and multivalent Sia and (GlcNAc)_n_ binder WGA exhibited significantly decreased NFIs in T2DM (all fold change = 0, *p* < 0.001). In addition, the *α*-Gal/GalNAc binder BS-I, (*β*-1,4)-linked GlcNAc binder DSA, and *α*- or *β*-linked terminal GalNAc binder SBA showed significantly decreased NFIs in T2DM (all fold change ≤ 0.60, *p* < 0.05) (Figure 2(b)).

Evaluating the alterations of urinary glycopatterns with the development of DN, the results showed that there were 6 lectins (e.g., the Sia*α*2-3Gal*β*1-4Glc(NAc)/Glc binder MAL-II, GalNAc and GalNAc*α*-1,3Gal binder PTL-I, and terminal in GalNAc and Gal binder SJA) that exhibited significantly increased NFIs in DN group I compared with HVs and T2DM, and high-Mannose, Man*α*1-6(Man*α*1-3)Man binder Con A showed significantly increased NFIs in DN group II compared with HVs and T2DM (*p* < 0.01). However, the *α*- or *β*-linked terminal GalNAc binder SBA and Fuc*α*-1,6GlcNAc binder PSA revealed significantly decreased NFIs in DN group II compared with HVs and T2DM (all fold change = 0, *p* < 0.001). The (GlcNAc)_2–4_ binder DSA and Sia*α*2-6Gal/GalNAc binder SNA exhibited significantly increased NFIs (all fold change ≥ 2.88, *p* < 0.05) in both DN groups compared with HVs and T2DM, and the Man*α*1-3Man binder GNA, *α*-Gal/GalNAc binder BS-I, *α*-Fuc binder AAL, and *α*- or *β*-linked terminal GalNAc binder SBA exhibited increased NFIs (all fold change ≥ 1.61, *p* < 0.05) in DN group I compared with T2DM. However, the (GlcNAc)_2–4_ binder STL exhibited significantly decreased NFIs in both DN groups compared with HVs and T2DM (all fold change = 0, *p* < 0.001). And the *α*-Gal/GalNAc binder BS-I and (GlcNAc)_n_ and branched (LacNAc)_n_ binder PWM showed decreased NFIs (fold change = 0.54, *p* < 0.05) in DN group II compared with HVs (showed in Figure 2(b)).

### 3.2. Alterations of Urinary Glycopatterns Associated with Different Degree of DN

Although the albuminuria and eGFR could reflect the process of DN, it also needs to find new sensitive biomarkers for monitoring the development of DN. The glycopatterns of urinary glycoproteins in different degree of DN were analyzed. The NFIs of each lectin from DN group I and group II were compared based on fold change (Table 2), and the results demonstrated that there were 9 lectins (e.g., the Sia*α*2-3Gal*β*1-4Glc(NAc)/Glc binder MAL-II, GalNAc and GalNAc*α*-1,3Gal binder PTL-I, and terminal in GalNAc and Gal binder SJA) that exhibited significantly decreased NFIs in DN group II compared with group I (all fold change = 0, *p* < 0.001). However, the high-Mannose, Man*α*1-6(Man*α*1-3)Man binder Con A showed significantly increased NFIs in DN group II compared with DN group I (*p* < 0.001) (showed in Figure 1(b)). Besides that, the Sia2-6Gal/GalNAc binder SNA showed significantly increased NFIs in DN group II compared with group I (fold change = 2.54, *p* < 0.01). The *α*-Gal/GalNAc binder BS-I, (GlcNAc)_n_ and branched (LacNAc)_n_ binder PWM, and Man*α*-1,3 man binder GNA exhibited significantly decreased NFIs (all fold change ≤ 0.55, *p* < 0.05) in DN group II compared with DN group I (Figure 2(b)).

### 3.3. Alteration of Glycopatterns between DN and NDRD

**Figure 3:**
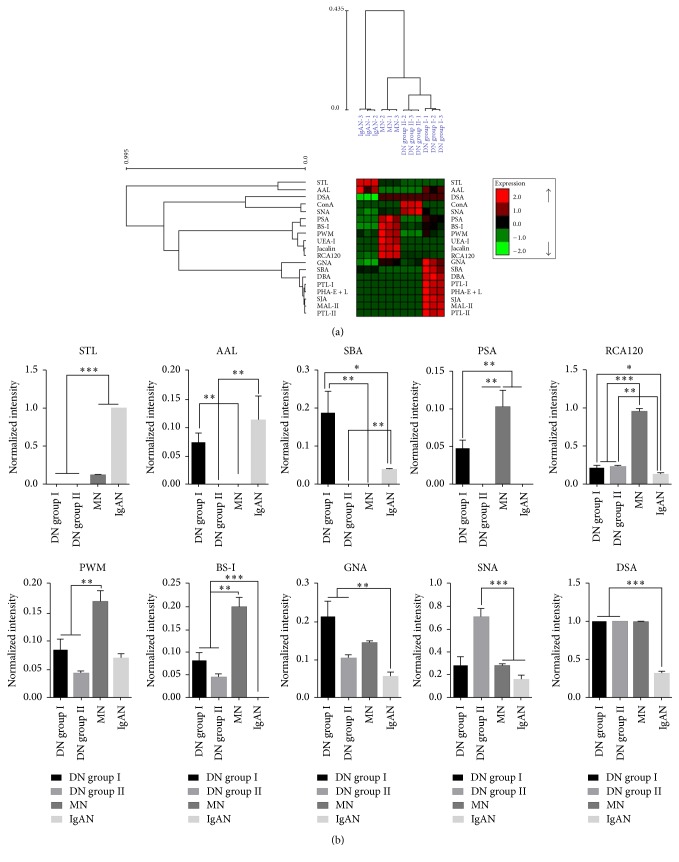
The alterations of glycopatterns between DN and NDRD. (a) Hierarchical clustering analysis of the 19 lectins with three biological replicates. DN groups and NDRD were listed in columns, and the lectins were listed in rows. (b) Ten lectins revealed significant differences between DN and NDRD. Significant differences between groups were analyzed according to Student's *t*-test, respectively (^*∗*^*p* < 0.05, ^*∗∗*^*p* < 0.01, and ^*∗∗∗*^*p* ≤ 0.001). The data were the averaged NFI ± SD of three biological replicates.

Currently, it is difficult to distinguish DN from NDRD in clinical practice. Renal biopsy is still a reliable clinical detection for diagnosis of DN. Here, the differences of urinary protein glycopatterns were investigated between DN (DN group I and DN group II) and NDRD (MN and IgAN). As a result of HCA, DN groups and NDRD were separated and lectins were categorized into four independent groups according to the NFIs of lectins, which revealed that the glycopatterns in urine showed difference between DN and NDRD (Figure 3(a)). The fold change between DN and NDRD groups was summarized in Table 3.

The results showed that there were 5 lectins (the Sia*α*2-3Gal*β*1-4Glc(NAc)/Glc binder MAL-II, GalNAc and GalNAc*α*-1,3Gal binder PTL-I, terminal in GalNAc and Gal binder SJA, GalNAc*α*1-3((Fuc*α*1-2))Gal binder DBA, and Gal and T-antigen binder PTL-II) to show high NFIs in DN group I and high-Mannose, Man*α*1-6(Man*α*1-3)Man binder ConA to show high NFIs in DN group II compared with NDRD (*p* < 0.001). However, the (GlcNAc)_2–4_ binder STL showed high NFIs in NDRD compared with DN groups, and *α*-Fuc binder AAL, *α*- or *β*-linked terminal GalNAc binder SBA and Fuc*α*-1,6GlcNAc binder PSA showed high NFIs in NDRD compared with DN groups II (*p* < 0.001). In addition, the Gal*β*1-3GalNAc*α*-Ser/Thr(T) binder Jacalin and Fuc*α*1-2Gal*β*1-4Glc(NAc) binder UEA-I exhibited high NFIs in MN compared with both DN groups (*p* < 0.001). Besides that, the *β*-D-GlcNAc binder DSA showed significantly higher NFIs in both DN groups than that in patients with IgAN (fold change = 3.28, *p* < 0.001), the Sia*α*2-6Gal/GalNAc binder SNA showed significantly increased NFIs in DN group II compared with NDRD (fold change ≥ 2.56, *p* < 0.001), and the Man*α*1-3Man binder GNA associated with increased NFIs in both DN groups compared with IgAN (fold change ≥ 1.85, *p* < 0.01). And the *β*-Gal/GalNAc binder RCA120 and *α*-Gal/GalNAc binder BS-I exhibited increased NFIs in both DN groups compared with patients with IgAN (fold change ≥ 1.74, *p* < 0.05) but showed decreased NFIs compared with MN (fold change ≤ 0.41, *p* < 0.05) However, the (GlcNAc)_n_ and branched (LacNAc)_n_ binder PWM exhibited significantly decreased NFIs (all fold change ≤ 0.50, *p* < 0.01) in both DN groups compared with MN (Figure 3(b)).

### 3.4. Assessment of Urinary Glycopatterns as Potential Biomarkers for Diagnosis of DN

**Figure 4:**
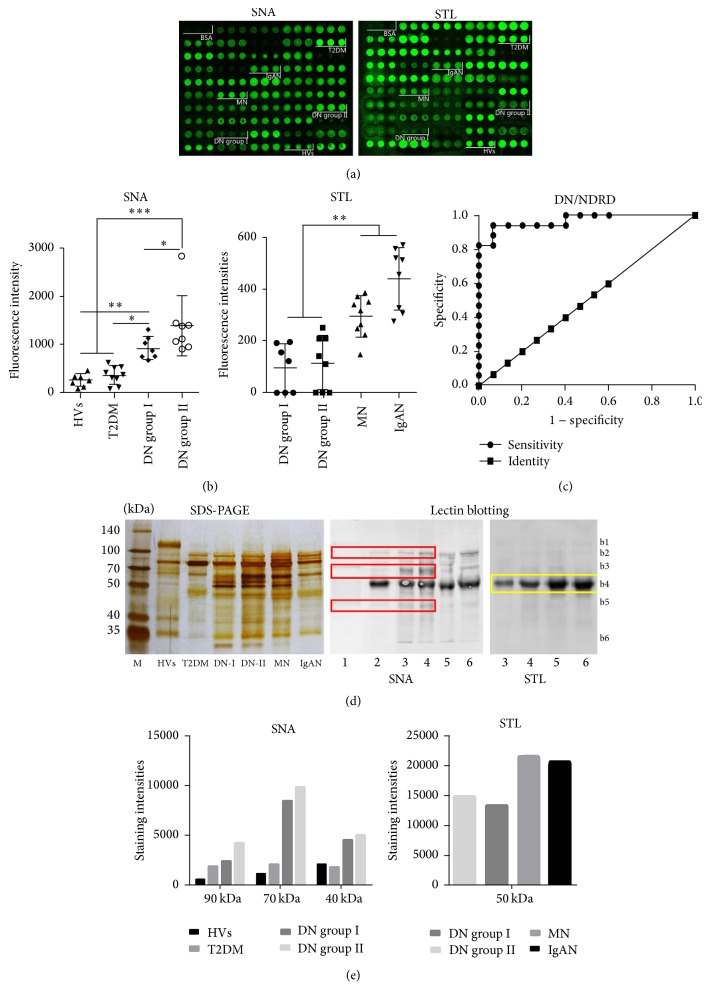
Validation of the differential expressions of the glycopatterns in the urine associated with DN. (a) Scan images of Cy3-labeled SNA and STL incubating to urinary protein microarray, respectively. The incubation images were extracted by GenePix 3.0. (b) Scatter plot analysis of the original data obtained from the urinary protein microarrays. The statistical significance of the differences between groups was calculated by Kruskal-Wallis test. (c) ROC curve analysis of STL to differentiate DN and NDRD, AUC = 0.968, and *p* < 0.0001. (d) SDS-PAGE and lectin blot of pooled urinary protein using SNA and STL, M, marker; lane 1, HVs; lane 2, patients with T2DM; lane 3, DN group I; lane 4, DN group II; lane 5, patients with MN; lane 6, patients with IgAN. (e) The lectin binding strength of selected protein bands is displayed, and the binding strength was counted by image J.

The most reliable way of biomarker discovery is to test the candidates with a large cohort of samples that have clear and sufficient clinical records. Here, a urinary protein microarray is produced by spotting 48 individual urine samples (7 HVs, 9 T2DM patients, 15 patients with DN (7 DN group I, 8 DN group II), and 17 patients with NDRD (9 MN and 8 IgAN)) on the surface of the epoxy slide. Each urine sample was spotted in triplicate, and the layout of the urinary protein microarrays was shown in Figure 4(a). SNA and STL that revealed significant differences (*p* < 0.05) in urinary glycopatterns according to the results of the lectin microarrays were selected to validate the alteration of the targeted glycopatterns in individual sample and evaluate the reliability of urinary glycopatterns as potential biomarkers for diagnosis of DN.

As a result, SNA staining showed increased signals in both DN groups compared with HVs and patients with T2DM and significant difference between DN group I and group II (*p* < 0.05). Interestingly, the signals of SNA staining showed an increasing trend with development of DN (Figure 4(b)). The result of correlation analysis indicated that the abundance of Sia*α*2-6Gal/GalNAc was positive correlation with the progression of DN (rs = 0.888). On the contrary, the signals of STL staining were significantly lower in both DN groups than that in NDRD (*p* < 0.001). ROC analysis showed that the ROC integral was 0.969 and *p* < 0.0001, which indicated the glycopattern of (GlcNAc)_2–4_ recognized by STL in urine glycoprotein could be a potential diagnostic indicator to differentiate DN and NDRD (Figure 4(c)). These results were consistent with lectin microarrays.

### 3.5. Lectin Blotting Analysis

In order to investigate precisely alterations of glycopatterns associated with DN, SDS-PAGE and lectin blotting analysis were performed with SNA and STL staining (Figure 4(d)). SNA staining showed obvious binding patterns with six distinct protein bands of approximately 120, 90, 70, 50, 40, and 25 kDa marked as b1–b6, respectively. The band of b4 showed strong staining signals in all samples except HVs. There were four bands (b2, b3, b5, and b6) to show increased signals in both DN groups compared with T2DM. Moreover, the glycoprotein bands (b2, b3, and b5) at about 90 kDa, 70 kDa, and 40 kDa exhibited increased staining signals in DN group II compared with group I (Figure 4(e)). Therefore, the abundance of Sia*α*2-6Gal exhibited general increase in urine with development of DN. Comparing DN and NDRD, the band of b4 (50 kDa) showed strong staining signals in both DN and NDRD groups but did not exhibit significant difference, whereas the bands of b3 (70 kDa) and b5 (40 kDa) revealed stronger staining intensities in DN groups than those in MN and IgAN.

STL staining showed obvious binding patterns with one distinct urinary protein band (b4) in both DN and NDRD at about 50 kDa. The binding signals of this glycoprotein band were significant higher in MN and IgAN than that in DN groups but have no difference between DN groups.

## 4. Discussion

Glycosylation is an important co- and posttranslational modification of glycoprotein, involved in many biological processes such as embryogenesis and neoplasm metastasis. The altered oligosaccharide moiety of glycoprotein was considered to be associated with the occurrence and development of diseases [26–29]. Abnormal glycosylation of certain glycoprotein in urine could be a more sensitive biomarker for disease diagnosis and monitoring. It is reported that aberrantly excreted urinary* O*-glycosylated proteins (clusterin, leucine-rich alpha-2-glycoprotein, and kininogen) could serve as potential biomarkers for the early detection of early stage ovarian cancer [30]. As a powerful tool, lectin microarray was used to investigate the glycopatterns of protein for decades. With the development of mass spectrometry, the glycan profile could be analysis by MS. However, as a rapid analysis approach, lectin microarray owned some unique advantages. First of all, the proteins do not need to enzymolysis prior to analysis, and the target is simple to prepare. Secondly, the linkage information of glycopatterns could be obtained by lectin microarray; however, mass spectrometry has difficulty in discriminating between the many structural isomers presented by glycans [31]. There is no denying that MS has advantage to acquire precisely information about the component of glycan. Notwithstanding intense efforts by multiple research groups, the use of mass spectrometry as a stand-alone technique for complete characterization of glycans is still far from complete [32].

In this study, glycopatterns of urine glycoprotein in patients with DN were compared with controls (healthy volunteers and patients with T2DM) and patients with NDRD (MN and IgAN), respectively. As a result, STL showed strong binding signals in HVs and patients with T2DM and IgAN; in addition, DSA exhibited strong binding signals in patients with DN and MN. It indicated that GlcNAc and polymers of GlcNAc were predominant glycopatterns in urine of patients. In urinary glycoprotein, Hex_5_HexNAc_4_-N-Asn was typically N-glycan observed which is similar to our findings [33]. As a result of lectin microarrays, there were 9 and 15 lectins to show binding signals in patients with T2DM and DN group I, which indicated more glycopatterns emerged in urine of patients in middle and advanced stage of DN without renal insufficiency (DN group I) compared with patients with T2DM. However, when compared to patients with overt proteinuria and renal insufficiency (DN group II), the situation was changed, only 7 lectins (e.g., WFA, GSL-II, and PNA) exhibited effective binding signals in DN group II, and 9 lectins (e.g., WFA, GSL-II, and PNA) failed to detect binding signals compared with DN group I. Furthermore, there were 3 lectins (BS-I, PWM, and GNA) to exhibit decreased NFIs in DN group II compared with DN group I. These findings demonstrated that the glycopatterns, especially the glycopatterns modified by Gal/GalNAc, were decreased as occurred in renal insufficiency. Inoue et al. reported the global reduction of the binding to lectins in urine samples of DN at macroalbuminuria stage [30]. Our finding further clarified that glycopatterns showed global reduction when occurring in renal insufficiency in stage of overt proteinuria. In rat diabetic nephropathy model which was induced by streptozotocin injection, the staining intensities of Gal/GalNAc binders PNA and RCA showed remarkable decrease in model after being induced for 5 weeks [34]. It is known that around 70% of the urinary proteins are estimated to be derived from the kidney and the urinary tract [35]. Therefore, we suspected that the general reduction in signature glycopatterns of Gal/GalNAc in kidney may contribute to the decrease of these glycopatterns in urinary glycoprotein with development of DN; the relationship between glycopatterns in kidney and urine will be clarified in next step.

In present study, we found that the glycopatterns of Sia*α*2-6Gal/GalNAc showed increased tendency with development of DN, and urinary protein microarray also confirmed this result. Our finding suggested Sia*α*2-6Gal/GalNAc is potential indicator for monitoring of DN. Furthermore, lectin blotting analysis was employed to investigate the precise alterations of Sia*α*2-6Gal/GalNAc. Generally, it showed higher staining signals in DN groups than that in HVs and T2DM. The glycoprotein bands at about 90 kDa, 70 kDa, and 40 kDa showed increased staining signals with development of DN. These results indicated the sialylation of these glycoproteins was increased and it may contribute to the upregulation of expression level of Sia*α*2-6Gal/GalNAc in urinary glycoprotein with development of DN. It is known that the level of serum sialic acid could indicate extensive vascular damage in T2DM and predict microvascular complications occurring in diabetics [36, 37]. As a result of our finding, the expression level of Sia*α*2-6Gal/GalNAc in urinary glycoprotein correlated with development of DN in stage of overt proteinuria. Similarly, it is reported that a 41 kDa of sialylated glycoprotein orosomucoid which contained Sia*α*2-6Gal/GalNAc increased excretion in DN, and it could be a potential biomarker for DN [38–40]. In addition, it is reported that the excretion of transferrin (76.5 kDa) and alpha-1-antitrypsin (46 kDa) showed marked increase in urine of patients with DN, which could reflect the development of microalbuminuria; moreover, urinary transferrin is a sensitive indicator of glomerular damage [41, 42]. Therefore, the glycopatterns of Sia*α*2-6Gal/GalNAc and glycoprotein modified by Sia*α*2-6Gal/GalNAc (especially the glycoproteins with molecular weight about 90 kDa, 70 kDa; and 40 kDa) in urine could be valuable potential diagnosis indicators for monitoring of DN, and these glycoproteins will be characterized by MS in future.

Although certain clinical manifestations such as shorter duration of diabetes, absence of retinopathy, presence of microscopic hematuria, and active urinary sediment are markers associated with NDRD in type 2 diabetes with clinical renal disease, and the histories of diabetes mellitus, systolic blood pressure, glycated HbA1c, hematuria, diabetic retinopathy, and hemoglobin are independently related to DN, renal biopsy remains indispensable to differentiate between DN and NDRD [43–48]. In this study, we examined the difference between DN and NDRD in urinary glycopatterns; our result showed that the relative abundance of (GlcNAc)_2–4_ in urinary glycoprotein could effectively distinguish between DN and NDRD, and ROC curve analysis also indicated that the glycopatterns recognized by STL have the potential to serve as a clinical predictor to distinguish between DN and NDRD. It demonstrated that the patients with IgAN exhibit circulating IgA1 with reduced Gal (galactose-deficient IgA1) [49–51]. It reported that deficiency of the *β*1,4GalT1 glycosyltransferase in genetic remodeling mouse could cause IgAN. Because of deficiency of the *β*1,4GalT1, *β*4-galactosylation of the* N*-glycans on the serum IgA was completely absent, which led to the exposure of GlcNAc of* N*-linked glycans [52]. In addition, urinary excretion of galactose-deficient IgA1 was elevated in patients with IgAN and the urinary Gd-IgA1 levels correlated with proteinuria [53]. Hiki et al. reported that* O*-glycans attached to serum IgA are truncated to expose GlcNAc, which correlates with antibody glomerular deposition in IgAN [54]. Therefore, we concluded that the exposure of GlcNAc on* N*-/*O*-glycans of IgA correlated with the increasement of (GlcNAc)_2–4_ in urinary glycoprotein from patients with IgAN.

In conclusion, we investigated the correlation of alterations of protein glycopatterns in urine samples from HVs, patients with T2DM, and patients with DN and NDRD. Our finding revealed that the elevated expression level of Sia*α*2-6Gal/GalNAc in urine was correlated with development of DN in stage of macroalbuminuria, and (GlcNAc)_2–4_ in urine could be a clinical predictor to distinguish between DN and NDRD. It should be pointed out that the sample size in this study is small, and more samples are being recruited to verify our findings and glycoprotein which associated with abnormal glycopatterns will be isolated and identified in next step. This study provided a new method to monitor the development of DN and distinguish between DN and NDRD based on the urinary glycopatterns analysis.

## Supplementary Material

The clinical information of individuals enrolled for urinary protein microarray were summarized in Table S1. The normalized fluorescent intensities for each lectin in HVs, T2DM, DN groups, MN and IgAN by the lectin microarray analysis based on data of 37 lectins were listed in Table S2.

## Figures and Tables

**Table 1:** Biochemical characteristics of clinical specimens.

	HVs	T2DM	DN (*n* = 19)		NDRD (*n* = 18)	
			DN group I	DN group II	MN	IgAN
Number of subjects	10	10	9	10	10	8
Age (years)	59.00 ± 3.05	58.50 ± 6.22	48.00 ± 7.45	52.60 ± 8.33	56.40 ± 6.64	53.63 ± 10.93
Sex (male/female)	4/6	5/5	4/5	8/2	6/4	4/4
BMI (kg/cm^2^)	24.2 ± 3.6	25.1 ± 2.9	26.0 ± 3.0	25.7 ± 3.2	27.2 ± 3.8	25.2 ± 3.0
Duration of diabetes (months)	∖	63.1 ± 15.5	126.2 ± 63.0	129.6 ± 56.4	69.6 ± 49.8	82.8 ± 50.2
Systolic blood pressure (mmHg)	119.7 ± 19.3	120.4 ± 21.4	152.1 ± 19.0	152.1 ± 19.0	136.4 ± 20.5	136.4 ± 20.5
Urinary protein (g/24 h)	∖	∖	1.41 ± 0.61	2.64 ±1.41	3.28 ± 1.56	2.84 ± 2.21
Creatinine (*µ*mol/L)	∖	63.70 ± 10.93	81.34 ± 14.87	140.83 ± 52.00	82.07 ± 29.88	114.70 ± 46.65
eGFR (ml · min^−1^ · 1.73 m^−2^)	∖	∖	78.68 ± 23.38	49.37 ± 18.64	83.99 ± 31.77	61.08 ± 30.57
HbA1c (%)	∖	6.6 ± 0.9	7.3 ± 1.6	7.2 ± 1.9	6.8 ± 1.0	7.0 ± 1.8
Diabetic retinopathy	∖	∖	5 (55.6%)	6 (60.0%)	3 (30%)	1 (12.5%)

**Table 2:** Fold change of the urinary glycopatterns based upon ratio of the NFIs of each lectin between HVs, T2DM, and DN groups.

Lectin name	Specificity		Fold change^a^				
		T2DM/HVs	DN group I/HVs	DN group I/T2DM	DN group II/HVs	DN group II/T2DM	DN group II/DN group I
MAL-II	Sia*α*2-3Gal*β*1-4Glc(NAc)/Glc	—	/^*∗∗∗*^	/^*∗∗∗*^	—	—	0^*∗∗∗*^

PTL-I	GalNAc, GalNAc*α*-1,3Gal, GalNAc*α*-1,3Gal*β*-1,3/4Glc	—	/^*∗∗∗*^	/^*∗∗∗*^	—	—	0^*∗∗∗*^

SJA	Terminal in GalNAc and Gal, anti-A and anti-B human blood group	—	/^*∗∗∗*^	/^*∗∗∗*^	—	—	0^*∗∗∗*^

AAL	*α*-Fuc, Fuc*α*1-6 GlcNAc (core fucose)	0^*∗∗∗*^	3.30^*∗∗*^	/^*∗∗∗*^	0^*∗∗∗*^	—	0^*∗∗∗*^

LEL	LacNAc and poly LacNAc, (GlcNAc)_n_	0^*∗∗∗*^	0^*∗∗∗*^	—	0^*∗∗∗*^	—	—

DBA	*α*GalNAc, Tn antigen, GalNAc*α*1-3((Fuc*α*1-2))Gal (blood group A antigen)	—	/^*∗∗∗*^	/^*∗∗∗*^	—	—	0^*∗∗∗*^

RCA120	*β*-Gal, Gal*β*-1,4GlcNAc (type II), Gal*β*1-3GlcNAc (type I)	0.799	1.05	1.31	1.17	1.46	1.12

STL	(GlcNAc)_2–4_, core (GlcNAc) of N-glycan	1	0^*∗∗∗*^	0^*∗∗∗*^	0^*∗∗∗*^	0^*∗∗∗*^	—

BS-I	*α*-Gal/GalNAc, Gal*α*-1,3Gal	0.60^*∗*^	0.97	1.61^*∗*^	0.54^*∗*^	0.89	0.55^*∗*^

ConA	High-Mannose, Man*α*1-6(Man*α*1-3)Man, terminal GlcNAc	—	—	—	/^*∗∗∗*^	/^*∗∗∗*^	/^*∗∗∗*^

PTL-II	Gal, blood group H, T-antigen	—	/^*∗∗∗*^	/^*∗∗∗*^	—	—	0^*∗∗∗*^

DSA	(*β*-1,4)-linked GlcNAc	0.21^*∗∗*^	2.88^*∗∗∗*^	13.61^*∗∗∗*^	2.88^*∗∗∗*^	13.61^*∗∗∗*^	1

SBA	*α*- or *β*-linked terminal GalNAc, (GalNAc)_n_, GalNAc*α*1-3Gal, blood-group A	0.32^*∗*^	1.16	3.63^*∗*^	0^*∗∗∗*^	0^*∗∗∗*^	0^*∗∗∗*^

PSA	Fuc*α*-1,6GlcNAc, *α*-D-Man, *α*-D-Glc	1.31	1.52	1.16	0^*∗∗∗*^	0^*∗∗∗*^	0^*∗∗∗*^

ACA	Gal*β*1-3GalNAc*α*-Ser/Thr (T antigen), sialyl-T(ST)	0^*∗∗∗*^	0^*∗∗∗*^	—	0^*∗∗∗*^	—	—

WGA	Multivalent Sia and (GlcNAc)_n_	0^*∗∗∗*^	0^*∗∗∗*^	—	0^*∗∗∗*^	—	—

PWM	(GlcNAc)_n_, branched (LacNAc)_n_	0.59	1.04	1.76	0.54^*∗*^	0.92	0.521^*∗*^

GNA	Man*α*1-3Man	1.46	2.39^*∗∗*^	1.64^*∗*^	1.17	0.80	0.488^*∗*^

PHA-E + L	Bisecting GlcNAc, biantennary N-glycans, tri- and tetraantennary complex-type N-glycan	—	/^*∗∗*^	/^*∗∗*^	—	—	0^*∗∗*^

SNA	Sia2-6Gal/GalNAc	1.37	4.34^*∗∗*^	3.16^*∗*^	11.03^*∗∗∗*^	8.05^*∗∗∗*^	2.54^*∗∗*^

^a^Fold changes of lectins bound to DN compared to healthy control and diabetic calculated with mean value of normalized florescence intensities of lectins obtained from 3 biological reduplicates. —, no significant difference; /, the denominator of the fold-change was infinite; 0, the numerator of the fold-change was zero. *∗* versus *p* < 0.05; *∗∗* versus *p* < 0.01; *∗∗∗* versus *p* < 0.001; *p* value was calculated by Student's *t*-test.

**Table 3:** Fold change of the urinary glycopatterns based upon ratio of the NFIs of each lectin between DN groups and NDRD.

Lectin name	Specificity	Fold change^a^			
		DN group I/MN	DN group I/IgAN	DN group II/MN	DN group II/IgAN
Jacalin	Gal*β*1-3GalNAc*α*-Ser/Thr(T), GalNAc*α*-Ser/Thr(Tn)	0^*∗∗∗*^	—	0^*∗∗∗*^	—

MAL-II	Sia*α*2-3Gal*β*1-4Glc(NAc)/Glc,	/^*∗∗∗*^	/^*∗∗∗*^	—	—

PTL-I	GalNAc, GalNAc*α*-1,3Gal, GalNAc*α*-1,3Gal*β*-1,3/4Glc	/^*∗∗∗*^	/^*∗∗∗*^	—	—

SJA	Terminal in GalNAc and Gal, anti-A and anti-B human blood group	/^*∗∗∗*^	/^*∗∗∗*^	—	—

AAL	*α*-Fuc, Fuc*α*1-6 GlcNAc (core fucose)	/^*∗∗*^	0.65	—	0^*∗*^

DBA	*α*GalNAc, Tn antigen, GalNAc*α*1-3((Fuc*α*1-2))Gal (blood group A antigen)	/^*∗∗∗*^	/^*∗∗∗*^	—	—

RCA120	*β*-Gal, Gal*β*-1,4GlcNAc (type II), Gal*β*1-3GlcNAc (type I)	0.22^*∗∗∗*^	1.74^*∗*^	0.24^*∗∗∗*^	1.95^*∗∗*^

STL	(GlcNAc)_2–4_, core (GlcNAc) of N-glycan	0^*∗∗∗*^	0^*∗∗∗*^	0^*∗∗∗*^	0^*∗∗∗*^

BS-I	*α*-Gal/GalNAc, Gal*α*-1,3Gal	0.41^*∗∗*^	/^*∗∗*^	0.22^*∗∗∗*^	/^*∗∗∗*^

ConA	High-Mannose, Man*α*1-6(Man*α*1-3)Man, terminal GlcNAc	—	—	/^*∗∗∗*^	/^*∗∗∗*^

PTL-II	Gal, blood group H, T-antigen	/^*∗∗∗*^	/^*∗∗∗*^	—	—

DSA	(*β*-1,4)-linked GlcNAc	—	3.28^*∗∗∗*^	—	3.28^*∗∗∗*^

SBA	*α*- or *β*-linked terminal GalNAc, (GalNAc)_n_, GalNAc*α*1-3Gal, blood-group A	/^*∗∗*^	4.64^*∗*^	—	0^*∗∗∗*^

PSA	Fuc*α*-1,6GlcNAc, *α*-D-Man, *α*-D-Glc	0.46^*∗*^	/^*∗∗*^	/^*∗∗*^	—

UEA-I	Fuc*α*1-2Gal*β*1-4Glc(NAc)	0^*∗∗∗*^	—	0^*∗∗∗*^	—

PWM	(GlcNAc)_n_, branched (LacNAc)_n_	0.50^*∗∗*^	1.20	0.26^*∗∗∗*^	0.62^*∗∗*^

GNA	Man*α*1-3Man	1.49	3.80^*∗∗*^	0.73	1.85^*∗∗*^

PHA-E + L	Bisecting GlcNAc, biantennary N-glycans, tri- and tetra-antennary complex-type N-glycan	/^*∗∗*^	—	—	—

SNA	Sia2-6Gal/GalNAc	1.00	1.80	2.56^*∗∗∗*^	4.57^*∗∗∗*^

^a^Fold changes of lectins bound to DN groups compared to NDRD calculated with mean value of normalized florescence intensities of lectins obtained from 3 biological reduplicates. —, no significant difference; /, the fold-change was infinite; 0, the numerator of the fold-change was zero. *∗* versus *p* < 0.05; *∗∗* versus *p* < 0.01; *∗∗∗* versus *p* < 0.001, *p* value was calculate by Student's *t*-test.

## References

[1] Locatelli F., Canaud B., Eckardt K.-U., Stenvinkel P., Wanner C., Zoccali C. (2003). The importance of diabetic nephropathy in current nephrological practice. *Nephrology Dialysis Transplantation*.

[2] Pisitkun T., Shen R.-F., Knepper M. A. (2004). Identification and proteomic profiling of exosomes in human urine. *Proceedings of the National Academy of Sciences of the United States of America*.

[3] Sun W., Li F., Wu S. (2005). Human urine proteome analysis by three separation approaches. *Proteomics*.

[4] Shao C., Li M., Li X. (2011). A tool for biomarker discovery in the urinary proteome: a manually curated human and animal urine protein biomarker database. *Molecular & Cellular Proteomics*.

[5] Kanauchi M., Akai Y., Hashimoto T. (2002). Transferrinuria in type 2 diabetic patients with early nephropathy and tubulointerstitial injury. *European Journal of Internal Medicine*.

[6] Currie G., Delles C. (2016). Urinary proteomics for diagnosis and monitoring of diabetic nephropathy. *Current Diabetes Reports*.

[7] Kim S. S., Song S. H., Kim I. J. (2013). Urinary cystatin C and tubular proteinuria predict progression of diabetic nephropathy. *Diabetes Care*.

[8] Araki S.-I., Haneda M., Koya D. (2013). Predictive effects of urinary liver-type fatty acid-binding protein for deteriorating renal function and incidence of cardiovascular disease in type 2 diabetic patients without advanced nephropathy. *Diabetes Care*.

[9] Panduru N. M., Forsblom C., Saraheimo M. (2013). Urinary liver-type fatty acid-binding protein and progression of diabetic nephropathy in type 1 diabetes. *Diabetes Care*.

[10] Dennis J. W., Granovsky M., Warren C. E. (1999). Glycoprotein glycosylation and cancer progression. *Biochimica et Biophysica Acta (BBA)—General Subjects*.

[11] Ohtsubo K., Marth J. D. (2006). Glycosylation in cellular mechanisms of health and disease. *Cell*.

[12] Hu S., Wong D. T. (2009). Lectin microarray. *PROTEOMICS—Clinical Applications*.

[13] Katrlík J., Gemeiner P., Šedivá A. (2013). Lectin microarray and surface plasmon resonance biochips for detection of glycosylation and glyco-biomarkers. *Current Opinion in Biotechnology*.

[14] Syed P., Gidwani K., Kekki H., Leivo J., Pettersson K., Lamminmäki U. (2016). Role of lectin microarrays in cancer diagnosis. *Proteomics*.

[15] Matsuda A., Kuno A., Nakagawa T. (2015). Lectin microarray-based sero-biomarker verification targeting aberrant O-linked glycosylation on Mucin 1. *Analytical Chemistry*.

[16] Liang Y., Ma T., Thakur A. (2015). Differentially expressed glycosylated patterns of *α*-1-antitrypsin as serum biomarkers for the diagnosis of lung cancer. *Glycobiology*.

[17] Selvin E., Rawlings A. M., Grams M. (2014). Fructosamine and glycated albumin for risk stratification and prediction of incident diabetes and microvascular complications: a prospective cohort analysis of the Atherosclerosis Risk in Communities (ARIC) study. *The Lancet Diabetes and Endocrinology*.

[18] Wang N., Xu Z., Han P., Li T. (2016). Glycated albumin and ratio of glycated albumin to glycated hemoglobin are good indicators of diabetic nephropathy in type 2 diabetes mellitus. *Diabetes/Metabolism Research and Reviews*.

[19] Fujimoto T., Miya M., Machida M. (2006). Improved recovery of human urinary protein for electrophoresis. *Journal of Health Science*.

[20] Tantipaiboonwong P., Sinchaikul S., Sriyam S., Phutrakul S., Chen S.-T. (2005). Different techniques for urinary protein analysis of normal and lung cancer patients. *Proteomics*.

[21] Magistroni R., Ligabue G., Lupo V. (2009). Proteomic analysis of urine from proteinuric patients shows a proteolitic activity directed against albumin. *Nephrology Dialysis Transplantation*.

[22] Qin Y., Zhong Y., Zhu M. J. (2013). Age- and sex-associated differences in the glycopatterns of human salivary glycoproteins and their roles against influenza A virus. *Journal of Proteome Research*.

[23] Pilobello K. T., Krishnamoorthy L., Slawek D., Mahal L. K. (2005). Development of a lectin microarray for the rapid analysis of protein glycopatterns. *ChemBioChem*.

[24] Kuno A., Ikehara Y., Tanaka Y. (2011). Multilectin assay for detecting fibrosis-specific glyco-alteration by means of lectin microarray. *Clinical Chemistry*.

[25] Tateno H., Toyota M., Saito S. (2011). Glycome diagnosis of human induced pluripotent stem cells using lectin microarray. *Journal of Biological Chemistry*.

[26] Block T. M., Comunale M. A., Lowman M. (2005). Use of targeted glycoproteomics to identify serum glycoproteins that correlate with liver cancer in woodchucks and humans. *Proceedings of the National Academy of Sciences of the United States of America*.

[27] Norton P. A., Comunale M. A., Krakover J. (2008). N-linked glycosylation of the liver cancer biomarker GP73. *Journal of Cellular Biochemistry*.

[28] Isailovic D., Kurulugama R. T., Plasencia M. D. (2008). Profiling of human serum glycans associated with liver cancer and cirrhosis by IMS-MS. *Journal of Proteome Research*.

[29] Dennis J. W., Granovsky M., Warren C. E. (1999). Glycoprotein glycosylation and cancer progression. *Biochimica et Biophysica Acta—General Subjects*.

[30] Mu A. K.-W., Lim B.-K., Hashim O. H., Shuib A. S. (2013). Identification of O-glycosylated proteins that are aberrantly excreted in the urine of patients with early stage ovarian cancer. *International Journal of Molecular Sciences*.

[31] Pilobello K. T., Slawek D. E., Mahal L. K. (2007). A ratiometric lectin microarray approach to analysis of the dynamic mammalian glycome. *Proceedings of the National Academy of Sciences of the United States of America*.

[32] Gupta G., Surolia A., Sampathkumar S.-G. (2010). Lectin microarrays for glycomic analysis. *OMICS A Journal of Integrative Biology*.

[33] Halim A., Nilsson J., Rüetschi U., Hesse C., Larson G. (2012). Human urinary glycoproteomics; attachment site specific analysis of N- and O-linked glycosylations by CID and ECD. *Molecular & Cellular Proteomics*.

[34] Ravidà A., Musante L., Kreivi M. (2015). Glycosylation patterns of kidney proteins differ in rat diabetic nephropathy. *Kidney International*.

[35] Thongboonkerd V., Malasit P. (2005). Renal and urinary proteomics: current applications and challenges. *Proteomics*.

[36] Prajna K., Ashok Kumar J., Rai S. (2013). Predictive value of serum sialic acid in type-2 diabetes mellitus and its complication (Nephropathy). *Journal of Clinical and Diagnostic Research*.

[37] Crook M. A., Pickup J. C., Lumb P. J., Georgino F., Webb D. J., Fuller J. H. (2001). Relationship between plasma sialic acid concentration and microvascular and macrovascular complications in type 1 diabetes: the EURODIAB complications study. *Diabetes Care*.

[38] Inoue K., Wada J., Eguchi J. (2013). Urinary fetuin-A is a novel marker for diabetic nephropathy in type 2 diabetes identified by lectin microarray. *PLoS ONE*.

[39] Bierhuizen M. F. A., De Wit M., Govers C. A. R. L. (1988). Glycosylation of three molecular forms of human *α*1-acid glycoprotein having different interactions with concanavalin A. Variations in the occurrence of di-, tri-, and tetraantennary glycans and the degree of sialylation. *European Journal of Biochemistry*.

[40] Jiang H., Guan G., Zhang R. (2009). Increased urinary excretion of orosomucoid is a risk predictor of diabetic nephropathy. *Nephrology*.

[41] Sharma K., Lee S., Han S. (2005). Two-dimensional fluorescence difference gel electrophoresis analysis of the urine proteome in human diabetic nephropathy. *Proteomics*.

[42] Moresco R. N., Sangoi M. B., De Carvalho J. A. M., Tatsch E., Bochi G. V. (2013). Diabetic nephropathy: traditional to proteomic markers. *Clinica Chimica Acta*.

[43] Ghani A. A., Al Waheeb S., Al Sahow A., Hussain N. (2009). Renal biopsy in patients with type 2 diabetes mellitus: indications and nature of the lesions. *Annals of Saudi Medicine*.

[44] Wilfred D., Mysorekar V., Venkataramana R., Eshwarappa M., Subramanyan R. (2013). Nondiabetic renal disease in type 2 diabetes mellitus patients: a clinicopathological study. *Journal of Laboratory Physicians*.

[45] Liang S., Zhang X.-G., Cai G.-Y. (2013). Identifying parameters to distinguish non-diabetic renal diseases from diabetic nephropathy in patients with type 2 diabetes mellitus: a meta-analysis. *PLoS ONE*.

[46] Zhou J., Chen X., Xie Y., Li J., Yamanaka N., Tong X. (2008). A differential diagnostic model of diabetic nephropathy and non-diabetic renal diseases. *Nephrology Dialysis Transplantation*.

[47] Sharma S. G., Bomback A. S., Radhakrishnan J. (2013). The modern spectrum of renal biopsy findings in patients with diabetes. *Clinical Journal of the American Society of Nephrology*.

[48] Liu M.-Y., Chen X.-M., Sun X.-F. (2014). Validation of a differential diagnostic model of diabetic nephropathy and non-diabetic renal diseases and the establishment of a new diagnostic model. *Journal of Diabetes*.

[49] Marth J. D., Grewal P. K. (2008). Mammalian glycosylation in immunity. *Nature Reviews Immunology*.

[50] Suzuki H., Fan R., Zhang Z. (2009). Aberrantly glycosylated IgA1 in IgA nephropathy patients is recognized by IgG antibodies with restricted heterogeneity. *Journal of Clinical Investigation*.

[51] Coppo R., Amore A. (2004). Aberrant glycosylation in IgA nephropathy (IgAN). *Kidney International*.

[52] Nishie T., Miyaishi O., Azuma H. (2007). Development of immunoglobulin A nephropathy-like disease in *β*-1,4-galactosyltransferase-I-deficient mice. *American Journal of Pathology*.

[53] Suzuki H., Allegri L., Suzuki Y. (2016). Galactose-deficient IgA1 as a candidate urinary polypeptide marker of IgA nephropathy?. *Disease Markers*.

[54] Hiki Y., Odani H., Takahashi M. (2001). Mass spectrometry proves under-O-glycosylation of glomerular IgA1 in IgA nephropathy. *Kidney International*.

