# Supplementary material for: Glycopatterns of Urinary Protein as New Potential Diagnosis Indicators for Diabetic Nephropathy
Source: J Diabetes Res. 2017 Mar 19;2017:5728087. doi: 10.1155/2017/5728087 (PMC5376433; doi:10.1155/2017/5728087)
Supplement: Supplementary file 1 — The clinical information of individuals enrolled for urinary protein microarray were summarized in Table S1. The normalized fluorescent intensities for each lectin in HVs, T2DM, DN groups, MN and IgAN by the lectin microarray analysis based on data of 37 lectins were listed in Table S2. [file 5728087.f1.docx]

**Table. S1** Clinical information of individuals enrolled for urinary protein microarray

| Category | Number | Age | Gender | Urinary protein (g/24h) | Creatinine (µmol/L) | eGFR (ml · min^−1^ · 1.73 m^−2^) |
| --- | --- | --- | --- | --- | --- | --- |
| Normal | 1 | 45 | Female | \ | \ | \ |
| Normal | 2 | 53 | Female | \ | \ | \ |
| Normal | 3 | 57 | Male | \ | \ | \ |
| Normal | 4 | 55 | Female | \ | \ | \ |
| Normal | 5 | 58 | Male | \ | \ | \ |
| Normal | 6 | 48 | Male | \ | \ | \ |
| Normal | 7 | 56 | female | \ | \ | \ |
| T2DM | 8 | 43 | Male | \ | 50 | \ |
| T2DM | 9 | 52 | Female | \ | 50 | \ |
| T2DM | 10 | 56 | Female | \ | 52.4 | \ |
| T2DM | 11 | 56 | Female | \ | 96.8 | \ |
| T2DM | 12 | 61 | Female | \ | 52 | \ |
| T2DM | 13 | 63 | Male | \ | 62.9 | \ |
| T2DM | 14 | 61 | Male | \ | 70.3 | \ |
| T2DM | 15 | 66 | Male | \ | 83.5 | \ |
| T2DM | 16 | 71 | Male | \ | 82.1 | \ |
| DN-I | 17 | 57 | Male | 0.93 | 78.2 | 88.72 |
| DN-I | 18 | 46 | Male | 0.18 | 85.6 | 83.49 |
| DN-I | 19 | 39 | Male | 1.25 | 67.8 | 112.98 |
| DN-I | 20 | 56 | Female | 0.27 | 86.3 | 58.97 |
| DN-I | 21 | 52 | Male | 0.45 | 47.9 | 159.14 |
| DN-I | 22 | 41 | Female | 1.20 | 43.9 | 137.04 |
| DN-I | 23 | 41 | Female | 2.50 | 76.3 | 72.41 |
| DN-II | 24 | 55 | Male | 3.64 | 150.3 | 42.05 |
| DN-II | 25 | 46 | Male | 2.80 | 111.8 | 61.99 |
| DN-II | 26 | 56 | Female | 1.50 | 128.2 | 37.35 |
| DN-II | 27 | 48 | Male | 5.07 | 116.1 | 43.21 |
| DN-II | 28 | 57 | Female | 3.25 | 119.5 | 40.36 |
| DN-II | 29 | 59 | Male | 1.80 | 239.0 | 24.27 |
| DN-II | 30 | 63 | Female | 2.20 | 126.2 | 37.13 |
| DN-II | 31 | 53 | Male | 3.00 | 210.3 | 28.75 |
| NDRD | 32 | 49 | Female | 1.26 | 58.2 | 95.46 |
| NDRD | 33 | 67 | Female | 2.52 | 72.9 | 88.06 |
| NDRD | 34 | 53 | Male | 0.56 | 97.4 | 69.73 |
| NDRD | 35 | 41 | Male | 2.48 | 61.0 | 126.35 |
| NDRD | 36 | 47 | Male | 0.45 | 75.4 | 96.23 |
| NDRD | 37 | 57 | Female | 3.00 | 73.6 | 70.60 |
| NDRD | 38 | 56 | Male | 4.50 | 50.6 | 147.15 |
| NDRD | 39 | 48 | Male | 1.80 | 93.9 | 74.39 |
| NDRD | 40 | 52 | Male | 2.29 | 150.6 | 42.43 |
| NDRD | 41 | 35 | Male | 1.33 | 88.6 | 84.81 |
| NDRD | 42 | 35 | Male | 1.96 | 133.2 | 52.98 |
| NDRD | 43 | 34 | Male | 1.51 | 148.1 | 47.16 |
| NDRD | 44 | 49 | Male | 0.12 | 83.6 | 84.70 |
| NDRD | 45 | 65 | Male | 4.32 | 79.4 | 84.88 |
| NDRD | 46 | 54 | Male | 0.53 | 83.2 | 83.51 |
| NDRD | 47 | 48 | Male | 1.21 | 178.1 | 35.54 |
| NDRD | 48 | 44 | Female | 0.60 | 63.5 | 88.23 |

**Table. S2** The normalized fluorescent intensities for each lectin in HVs, T2DM, DN groups, MN and IgAN by the lectin microarray analysis based on data of 37 lectins*^a^*

| Lectin | HVs | T2DM | DN group I | DN group II | MN | IgAN |
| --- | --- | --- | --- | --- | --- | --- |
| Jacalin | / | / | / | / | 0.071 ± 0.002 | / |
| ECA | / | / | / | / | / | / |
| HHL | / | / | / | / | / | / |
| WFA | / | / | / | / | / | / |
| GSL-II | / | / | / | / | / | / |
| MAL-II | / | / | 0.042 ± 0.004 | / | / | / |
| PHA-E | / | / | / | / | / | / |
| PTL-I | / | / | 0.050 ± 0.008 | / | / | / |
| SJA | / | / | 0.058 ± /0.006 | / | / | / |
| PNA | / | / | / | / | / | / |
| EEL | / | / | / | / | / | / |
| AAL | 0.022 ± 0.008 | / | 0.073 ± 0.017 | / | / | 0.113 ± 0.043 |
| LTL | / | / | / | / | / | / |
| MPL | / | / | / | / | / | / |
| LEL | 0.026 ± 0.003 | / | / | / | / | / |
| GSL-I | / | / | / | / | / | / |
| DBA | / | / | 0.053 ± 0.007 | / | / | / |
| LCA | / | / | / | / | / | / |
| RCA120 | 0.200 ± 0.043 | 0.160 ± 0.015 | 0.209 ± 0.026 | 0.234 ± 0.009 | 0.956 ± 0.040 | 0.120 ± 0.025 |
| STL | 1 ± 0 | 1 ± 0 | / | / | 0.129 ± 0.006 | 1 ± 0 |
| BS-I | 0.081 ± 0.018 | 0.051 ± 0.004 | 0.082 ± 0.017 | 0.045 ± 0.006 | 0.199 ± 0.022 | / |
| ConA | / | / | / | 0.027 ± 0.001 | / | / |
| PTL-II | / | / | 0.041 ± 0.004 | / | / | / |
| DSA | 0.348 ± 0.057 | 0.074 ± 0.008 | 1 ± 0 | 1 ± 0 | 0.996 ± 0.006 | 0.305 ± 0.040 |
| SBA | 0.162 ± 0.047 | 0.052 ± 0.006 | 0.189 ± 0.058 | / | / | 0.041 ± 0.002 |
| VVA | / | / | / | / | / | / |
| NPA | / | / | / | / | / | / |
| PSA | 0.031 ± 0.007 | 0.041 ± 0.005 | 0.047 ± 0.011 | / | 0.102 ± 0.022 | / |
| ACA | 0.017 ± 0.003 | / | / | / | / | / |
| WGA | 0.043 ± 0.008 | / | / | / | / | / |
| UEA-I | / | / | / | / | 0.066 ± 0.013 | / |
| PWM | 0.081 ± 0.014 | 0.048 ± 0.016 | 0.085 ± 0.020 | 0.044 ± 0.003 | 0.169 ± 0.020 | 0.070 ± 0.008 |
| MAL-I | / | / | / | / | / | / |
| GNA | 0.089 ± 0.016 | 0.131 ± 0.012 | 0.214 ± 0.041 | 0.105 ± 0.009 | 0.143 ± 0.008 | 0.056 ± 0.011 |
| BPL | / | / | / | / | / | / |
| PHA-E+L | / | / | 0.041 ± 0.009 | / | / | / |
| SNA | 0.065 ± 0.010 | 0.089 ± 0.004 | 0.280 ± 0.079 | 0.7131 ± 0.071 | 0.279 ± 0.015 | 0.1561 ± 0.041 |

*^a^*Normalized fluorescent intensities (NFI) obtained for three repeated slides were averaged and its SD was counted; /, negative signals. HVs, healthy volunteers; T2DM, type 2 diabetic mellitus; DN diabetic nephropathy; MN, membranous nephropathy; IgAN, IgA nephropathy.
